# Extreme drought along the tropic of cancer (Yunnan section) and its impact on vegetation

**DOI:** 10.1038/s41598-024-58068-w

**Published:** 2024-03-29

**Authors:** Yanke Zhang, Tengfei Gu, Suling He, Feng Cheng, Jinliang Wang, Hui Ye, Yunfeng Zhang, Huai Su, Qinglei Li

**Affiliations:** 1 Faculty of Geography, Yunnan Normal University, Kunming, 650500 China; 2 Key Laboratory of Resources and Environmental Remote Sensing for Universities in Yunnan Kunming, Kunming, 650500 China; 3 Center for Geospatial Information Engineering and Technology of Yunnan Province, Kunming, 650500 China; 4 Wuhan Tianjihang Information Technology Co., Ltd., Wuhan, 430074 China; 5 Badong National Observation and Research Station of Geohazards, China University of Geosciences (Wuhan), Wuhan, 430074 China; 6 School of Life Sciences, Yunnan Normal University, Kunming, 650500 China

**Keywords:** Extreme drought, Vegetation, MODIS, Vegetation recovery, Yunnan, Climate sciences, Ecology, Natural hazards

## Abstract

The frequent occurrence of extreme weather events is one of the future prospects of climate change, and how ecosystems respond to extreme drought is crucial for response to climate change. Taking the extreme drought event in the Tropic of Cancer (Yunnan section) during 2009–2010 as a case study, used the standardized precipitation evapotranspiration index to analyse the impact of extreme drought on enhanced vegetation index (EVI), leaf area index (LAI) and gross primary productivity (GPP), and to analyzed the post extreme drought vegetation recovery status. The results indicate the following: (1) Due to the cumulative effects of drought and vegetation phenology, vegetation growth in the months of March to May in 2010 was more severely affected. (2) Compared to EVI and LAI, GPP is more sensitive to drought and can accurately indicate areas where drought has impacted vegetation. (3) Following an extreme drought event, 70% of the vegetation can recover within 3 months, while 2.87–6.57% of the vegetation will remain unrecovered after 6 months. (4) Cropland and grassland show the strongest response, with longer recovery times, while woodland and shrubland exhibit weaker responses and shorter recovery times. This study provides a reference for the effects of extreme drought on vegetation.

## Introduction

The intergovernmental panel on climate change (IPCC) Sixth Assessment Report points out that the frequent and intense occurrence of extreme climate events is one of the important features of global climate change in recent years^1^. Among them, extreme drought is the extreme climate event with the largest impact range, the longest lasting effect and the most serious loss to human beings. Compared with ordinary drought, extreme drought causes serious water imbalance in plants, which not only inhibits physiological processes such as photosynthesis and respiration but also inhibits plant growth and development and even causes death^2^. It may also have more serious, lasting, or even irreversible impacts on the composition, structure, and function of terrestrial ecosystems through processes such as regulating plant-microbial relationships and changing community composition^3–5^. Compared with the effects on resilient ecological environments, those on fragile ecological environments affected by extreme drought are more profound and obvious.

Yunnan Province is a leading province in China's ecological civilization construction and is one of the regions with the richest biodiversity in the world^6–8^. At the same time, Yunnan Province is also a region with a high incidence of drought. From 1950 to 2014, severe drought occurred every two to three years^9^, and the drought from autumn 2009 to spring 2010 was the most severe since records have been kept by the Yunnan Provincial Meteorological Bureau since 1959. This drought caused many small and medium-sized rivers to be cut off and reservoirs to dry up, which had a serious impact on the ecological environment of the region^10^, such as large-scale crop disasters^11^, the death of precious wild plants^12^ and water quality changes in plateau lakes^13^. Many studies have been conducted on the impact of the drought event on vegetation during autumn 2009 to spring 2010. It is generally agreed that extreme drought significantly reduces the vegetation index (Normalized Difference Vegetation Index (NDVI), Enhanced Vegetation Index (EVI)) and productivity level (Gross Primary Productivity (GPP), Net Primary Production (NPP)) in Yunnan Province^14–17^, and there are significant differences in the response of different vegetation types to extreme drought^18,19^. However, the existing research lacks the vegetation recovery status after the drought event, as well as the difference in the order and degree of response of different vegetation-related indicators to the drought event.

Combined with previous studies, we intend to take the Tropic of Cancer (Yunnan section) region as the study area and use standardized precipitation evapotranspiration index (SPEI), meteorological station data (temperature, precipitation), EVI, leaf area index (LAI), GPP and other data to analyse the impact of this extreme drought on vegetation and the time needed for subsequent vegetation recovery. This study aims to answer the following questions: (1) Are there any differences in the responses of EVI, LAI and GPP to this drought event? (2) How long does it take for vegetation to return to normal levels after an extreme drought? (3) Is there any difference in the recovery time of different vegetation types?

## Materials and methods

### Study area

**Figure 1:**
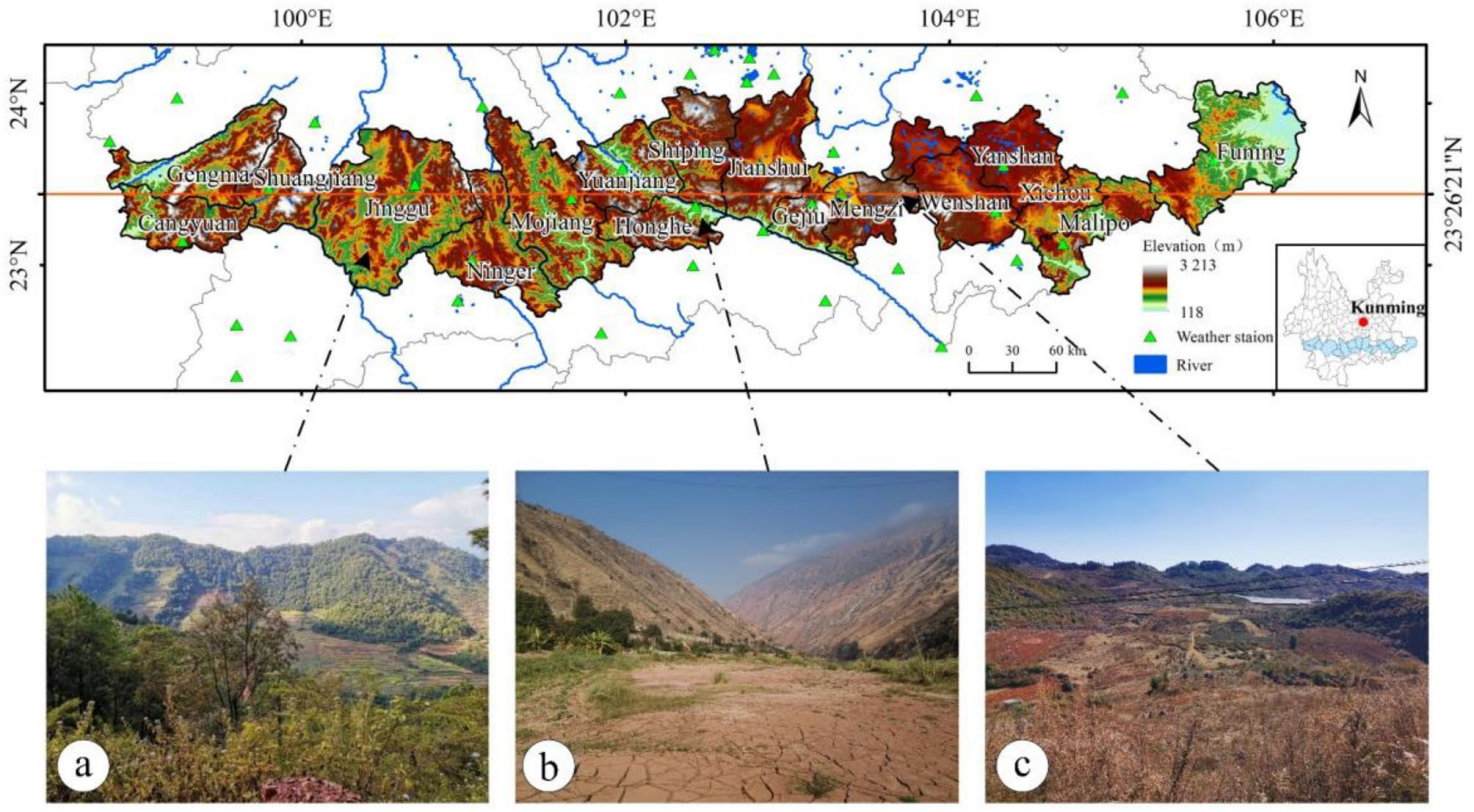
Location map of the Tropic of Cancer (Yunnan section): (**a**) Mountain Forest; (**b**) Yuanjiang-Honghe Hot-dry River Valley and (**c**) Karst Scenerys—created using ArcGIS v10.2^22^.

We selected the counties and cities on the Tropic of Cancer in Yunnan Province as the study area (referred to as the Tropic of Cancer (Yunnan section)) (Fig. 1), including Funing, Xichou, Malipo, Yanshan and 17 other counties (The map was created using ArcGIS 10.2 software (http://www.arcgis.com), and the following maps were made using the same software.). The geographical range is 98° 48′ 40″–106° 11′ 39″ E, 22° 48′ 54″–24° 10′ 44″ N, and the total area is 56 591.77 km^2^. This region is characterized by low latitude, abundant meteorological zones and rich geomorphology. Topographically, the region includes the western longitudinal Range-valley region, the middle Yunnan Plateau and the East Yunnan Plateau^20^. The region is dominated by the plateau subtropical monsoon climate, while there are more localized microclimates. In addition, the region is not divided into four seasons, but the dry and wet seasons are obvious. In summary, the unique climate and geomorphic conditions cause vegetation growth to have obvious regional characteristics^21^, ranging from tropical rainforest to shrub.

### Data sources

#### MODIS data and preprocessing

We used MODIS EVI, MODIS LAI and MODIS GPP data in this research, the time range of the data provided is from 2007 to 2013, and their basic information is presented in Table 1. The source of the above data is http://ladsweb.nascom.nasa.gov. The data preprocessing includes outlier removal, data quality improvement, monthly data synthesis, and data downsampling to 1 km.

**Table 1:** Introduction of MODIS data.

Product ID	Layer name	Unit	Time resolution	Effective range	Scale factor
MOD13Q1	250m_16_days_EVI	–	16d	− 2000–10,000	0.0001
MOD15A2H	LAI_500m	m^2 ^m^−2^	8d	0–100	0.1
MOD17A2H	GPP_500m	kg C/m^2^	1 m	0–30,000	0.0001

#### Meteorological data and preprocessing

The meteorological data in this research are temperature and precipitation data from 125 meteorological stations in Yunnan Province. The data are from the Data Center for Resources and Environmental Sciences, Chinese Academy of Sciences (http://www.resdc.cn). To facilitate subsequent analysis, the above data should be combined into monthly data, using the average temperature of the month as the monthly temperature and the total precipitation of the month as the monthly precipitation.

### Methods

#### Identification of extreme drought time

The SPEI is a drought index based on monthly precipitation and temperature data^23^, which preserves standardized precipitation index (SPI) sensitivity to precipitation and Palmer drought severity index (PDSI) sensitivity to evapotranspiration^24^. The SPEI includes several time scales, among which SPEI-1 is the monthly dry and wet level, SPEI-3 is the seasonal dry and wet level, SPEI-6 is the semiannual dry and wet level, and SPEI-12 is the annual dry and wet level. We used the 3-month SPEI (indicated as SPEI-3) to characterize the 2009–2010 drought event, as this temporal length of SPEI has been proved for its capacity in well capturing the characteristics of short-term variations of soil moisture condition^15^.

1. *Calculation of SPEI-3:* We used the SPEI-R package provided by the National Center for Atmospheric Research (NCAR) to calculate the SPEI. Meanwhile, to analyse the spatial distribution of drought, we used ANUSPLIN to interpolate the SPEI-3 data of 125 meteorological stations in Yunnan Province, in which the spline frequency was set to 2, the covariate was DEM elevation data, and the spatial resolution of the interpolation was 1 km. Then, the SPEI-3 spatial interpolation map of Yunnan Province is clipped by the vector map of the study area.
2. *Calculation of standardized anomaly index:* We used the standardized anomaly index to calculate the anomaly of SPEI-3^15^, which was used to analyse the spatial extent, duration, severity, onset and end time of extreme drought in the Tropic of Cancer (Yunnan Section) during 2009–2010. The formula is as follows:
  1 $$ SA_{SPEI - 3} = \frac{{SPEI_{3}^{i,t} - \overline{{SPEI_{3}^{i,t} }} }}{{\sigma \left( {SPEI_{3}^{i,t} } \right)}}\left( {t = 1,2, \ldots ,12} \right) $$

In Formula (1), $SPEI_{3}^{i,t}$ is the value of SPEI-3 on pixel *i* in middle *t* from 2009 to 2010, $\overline{{SPEI_{3}^{i,t} }}$ is the mean of SPEI-3 on pixel *i* in the *t* from 2001 to 2020, and $\sigma \left( {SPEI_{3}^{i,t} } \right)$ is the standard deviation value of SPEI-3 on pixel *i* in the *t* from 2001 to 2020. It is worth noting that SA_SPEI-3_ is a unitless value that represents the degree to which the SPEI-3 value deviates from the normal value for month *t*.

In addition, to measure the changes in temperature and precipitation during extreme drought events, we use the standardized anomaly index to calculate the standardized anomalies of temperature and precipitation, expressed as SA_Pre_ and SA_Tmp_, respectively. The formula is as follows:

2 $$ SA_{Pre} = \frac{{pre_{{}}^{i,t} - \overline{{pre_{{}}^{i,t} }} }}{{\sigma \left( {pre_{{}}^{i,t} } \right)}}\left( {t = 1,2, \ldots ,12} \right) $$

3 $$ SA_{Tmp} = \frac{{tmp_{{}}^{i,t} - \overline{{tmp_{{}}^{i,t} }} }}{{\sigma \left( {tmp_{{}}^{i,t} } \right)}}\left( {t = 1,2, \ldots ,12} \right) $$

In Formulas (2) and (3), $pre^{i,t}$ and $tmp^{i,t}$ represent the temperature and precipitation values of pixel *i* in month *t*, respectively. $\overline{{pre^{i,t} }}$,$\overline{{tmp^{i,t} }}$,$\sigma \left( {pre^{i,t} } \right)$ and $\sigma \left( {tmp^{i,t} } \right)$ represent the mean temperature, mean precipitation, standard deviation of temperature and standard deviation of precipitation on pixel *i* in month *t*, respectively.

**Figure 2:**
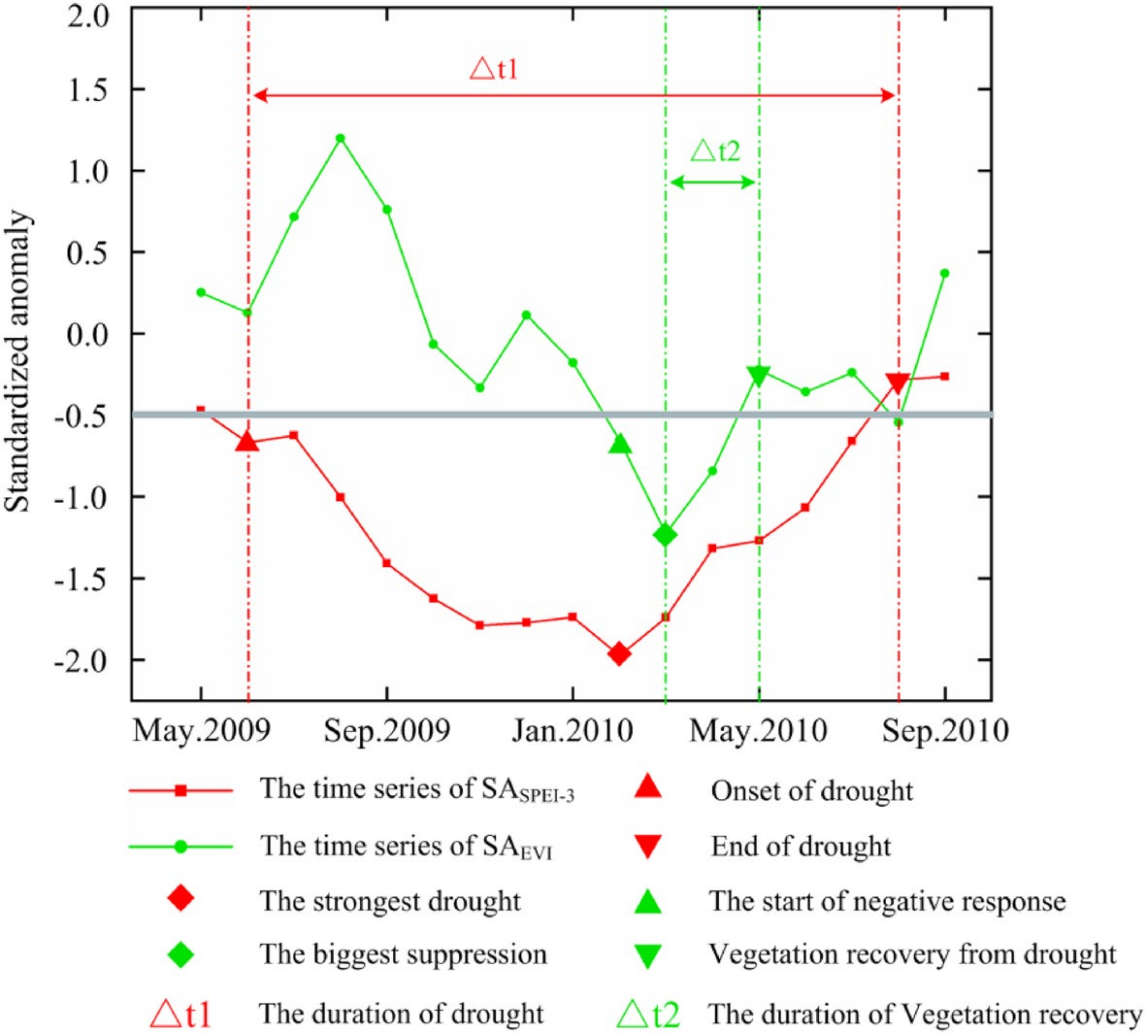
Schematic diagram of recognition of drought and its impact on vegetation.

(3) *Identification of extreme drought:* Fig. 2 shows a schematic diagram that describes the main time parameters of drought on each grid. Referring to Saft et al.'s method^25^, we smoothed SA_SPEI-3_ using a three-month average to avoid the unreasonable interruption of a one-month rainy season to a long and continuous dry period. We used smoothed SA_SPEI-3_ to identify the beginning and end periods of drought events. The beginning of the drought period is defined as follows: the first month SA_SPEI-3_ is below the threshold (− 0.5) (indicated by a red equilateral triangle in Fig. 2), the end of the drought period is defined as follows: the first month after the most intense drought SA_SPEI-3_ is above the threshold (− 0.5) (indicated by a red inverted triangle in Fig. 2), the duration of the drought is the interval between the beginning and the end of the drought (which is △t1 in Fig. 2), and the drought intensity is the value of SA_SPEI-3_.

#### Analysis of the impact of extreme drought on vegetation

1. *Calculation of standardized anomaly index of vegetation index:* To check the response of vegetation after drought, we used the standardized anomaly index of EVI, LAI and GPP to analyse vegetation changes during drought^26,27^, and the formula is as follows:
  4 $$ SA_{Veg} = \frac{{x^{i,t} - \overline{{x^{i,t} }} }}{{\sigma \left( {x^{i,t} } \right)}}\left( {t = 1,2, \ldots ,12} \right) $$

In Formula (4), $x^{i,t}$ represents the values of EVI, LAI and GPP on pixel *i* in month *t* of 2009–2010, $\overline{{x^{i,t} }}$ represents the mean value of EVI, LAI and GPP on pixel *i* in month *t* during the reference period (2007–2013), and $\sigma \left( {x^{i,t} } \right)$ represents the standard deviation value of EVI, LAI and GPP on pixel *i* in month *t* during the reference period (2007–2013). There are two reasons for choosing 2007–2013 as the reference years. First, there were almost no drought events in 2007–2008 and 2012–2013, which can be defined as normal years. Second, the above years are adjacent years from 2009 to 2010, which indicates that the degree of vegetation affected by human factors is not much different from the above years.

As shown in Fig. 2 (using EVI as an example), we define the first month with SA_EVI_ below -0.5 as the beginning of the period in which vegetation is negatively affected by drought (represented by a green regular triangle in Fig. 2) and define the month with the minimum value of SA_EVI_ as the month in which vegetation is most affected by drought (represented by a green diamond in Fig. 2). This month also represents the beginning of vegetation recovery from drought, and as the drought intensity decreases, the vegetation will gradually regain its vitality. We define the first month with SA_EVI_ above − 0.5 as the end of vegetation recovery from drought (represented by the green inverted triangle in Fig. 2). Based on these factors, we can calculate the time needed for vegetation recovery (represented by △t2 in Fig. 2).

(2) *Analysis of correlation coefficient:* We used the Pearson correlation coefficient to analyse the response of vegetation to extreme drought in 2009–2010^28^. Considering the lag of vegetation response to drought, we use the mean of correlation coefficients over six periods, including the current month, lag one month, two months, three months, four months, and five months, as the final correlation coefficient between SA_EVI_, SA_LAI_, SA_GPP_ and SA_SPEI-3_.
  5 $$ r_{mean} = mean\left( {r_{i} } \right)\quad 0 \le {\text{i}} \le {5} $$

In Formula (5), *r*_*i*_ represents the Pearson correlation coefficient with a lag of *i* months; when *i* is 0, there is no lag effect, and when *i* is 1 to 5, there is a lag of 1 to 5 months.

## Results

### Drought events in 2009–2010

**Figure 3:**
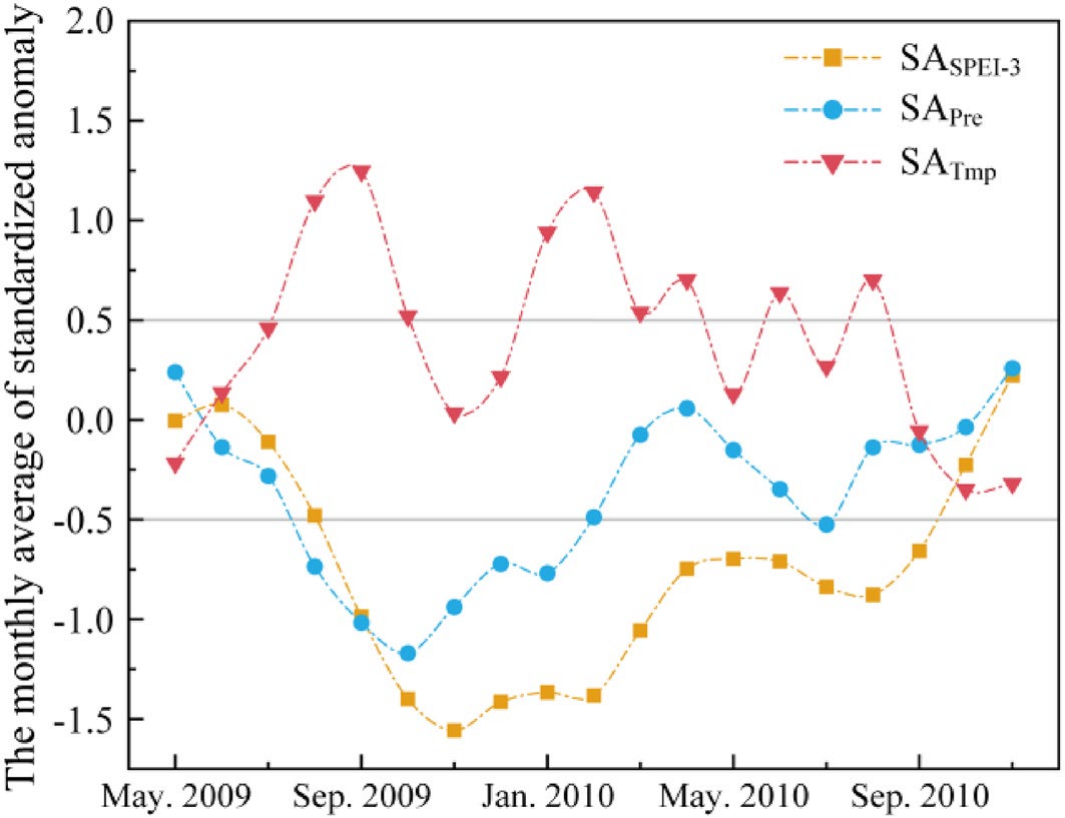
SA_SPEI-3_, SA_Pre_ and SA_Tmp_ from May 2009 to November 2010.

Figure 3 shows SA_SPEI-3_, SA_Pre_ and SA_Tmp_ in the study area from May 2009 to November 2010. The occurrence of drought is mainly concentrated between September 2009 and September 2010. There were nine months with SA_Tmp_ above 0.5, which indicated that there was an obvious warming phenomenon during this period, and there were six months with SA_Pre_ below 0.5, indicating that there was less precipitation during this period.

**Figure 4:**
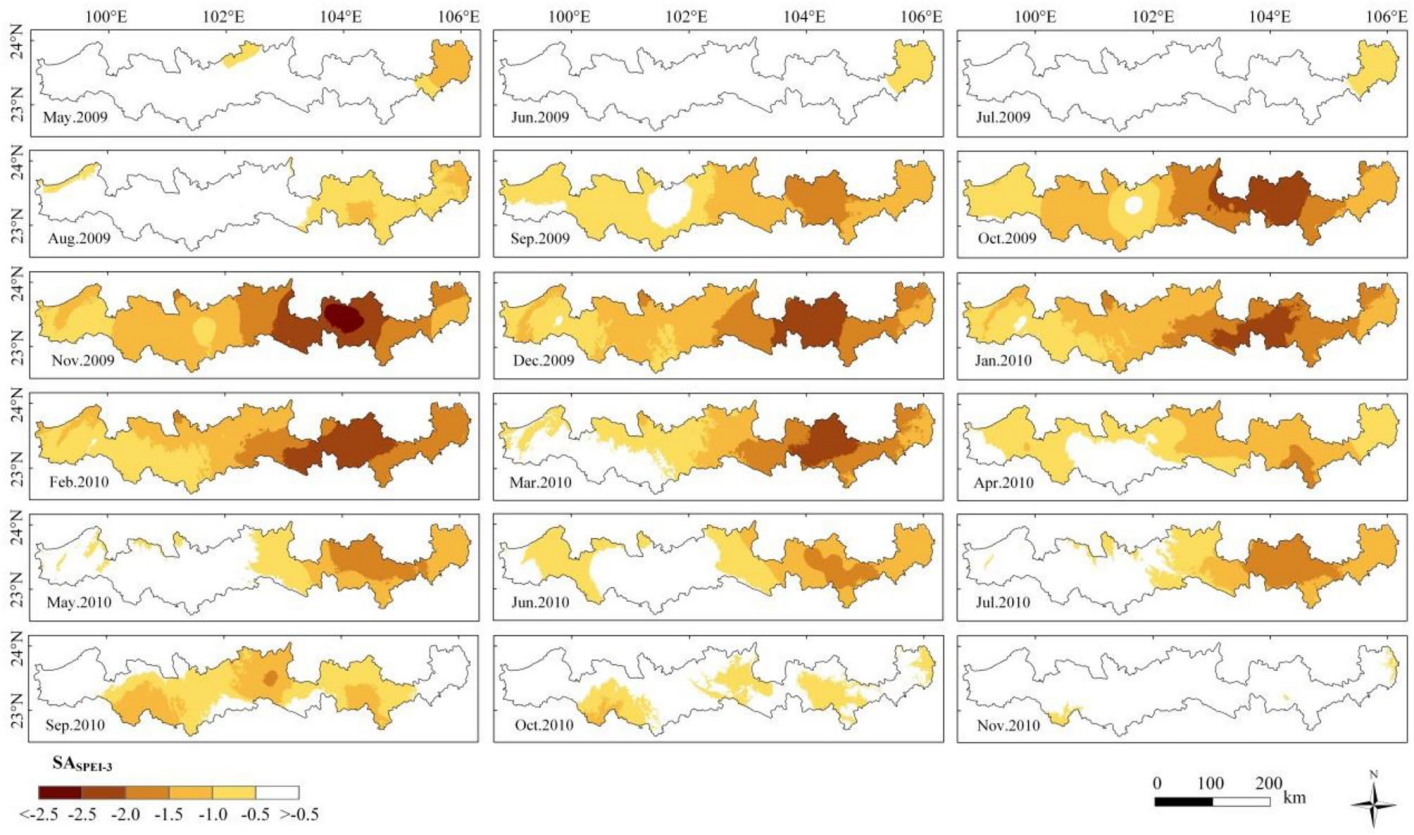
Spatial distribution of SA_SPEI-3_ from May 2009 to November 2010—created using ArcGIS v10.2^22^.

Figure 4 shows the spatial distribution of SA_SPEI-3_ in the study area. From October 2009 to February 2010, drought fully broke out in the study area, reaching almost 100%. In this period, the most serious month was November 2009, and the average value of SA_SPEI-3_ reached − 1.57. From October 2009 to March 2010, SA_SPEI-3_ reached − 2 in some parts of the Tropic of Cancer (Yunnan Section), which indicated that extreme drought occurred.

**Figure 5:**
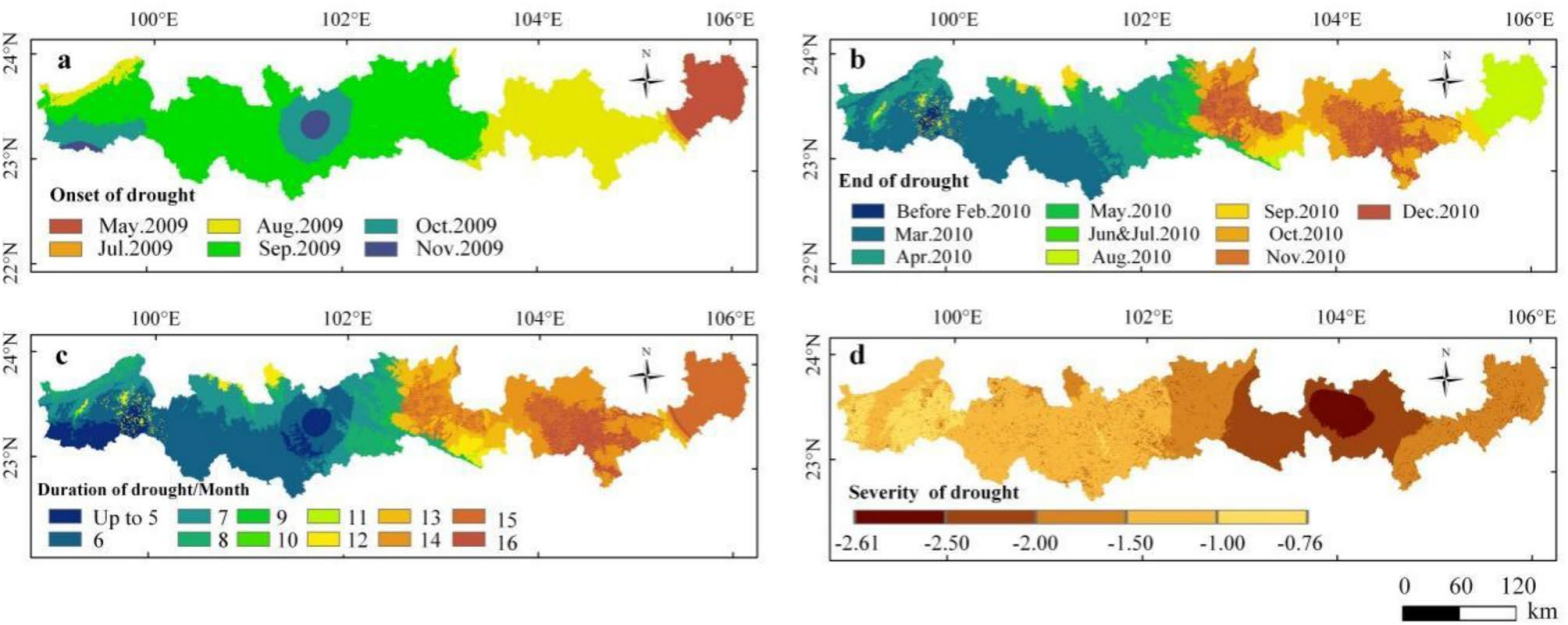
Statistics of drought events from 2009 to 2010 (**a**: onset of drought; **b**: end of drought; **c**: duration of drought; **d**: severity of drought) —created using ArcGIS v10.2^22^.

Figures 4 and 5 show that there was obvious spatial heterogeneity in the onset, end, duration and severity of this drought event. In May 2009, Funing County in the eastern part of the study area was the first county to experience drought. In September, a large area of drought occurred in the western part of the study area. From October to November, drought occurred in the whole study area. In April 2010, the drought ended in the western longitudinal mountain valley area, and in August 2010, the drought ended in Funing County. The drought did not completely end until September–December 2010 in the east-central region. In summary, drought began early and ended late in the eastern and central parts of the study area, with a duration of approximately 11–16 months. Meanwhile, the drought began late and ended early in the western longitudinal range-valley region, with a duration of approximately 5–7 months. In terms of the degree of drought, the most serious were Gejiu city, Mengzi city, Wenshan County, Yanshan County and Xichou County (SA_SPEI-3_ < − 2), the more serious were Funing County and Malipo County (SA_SPEI-3_ < − 1.5), and the slightly serious was the western longitudinal mountain valley area (SA_SPEI-3_ < − 1).

### Analysis of drought response of different vegetation indices

#### Analysis of the EVI standardized anomaly index

**Figure 6:**
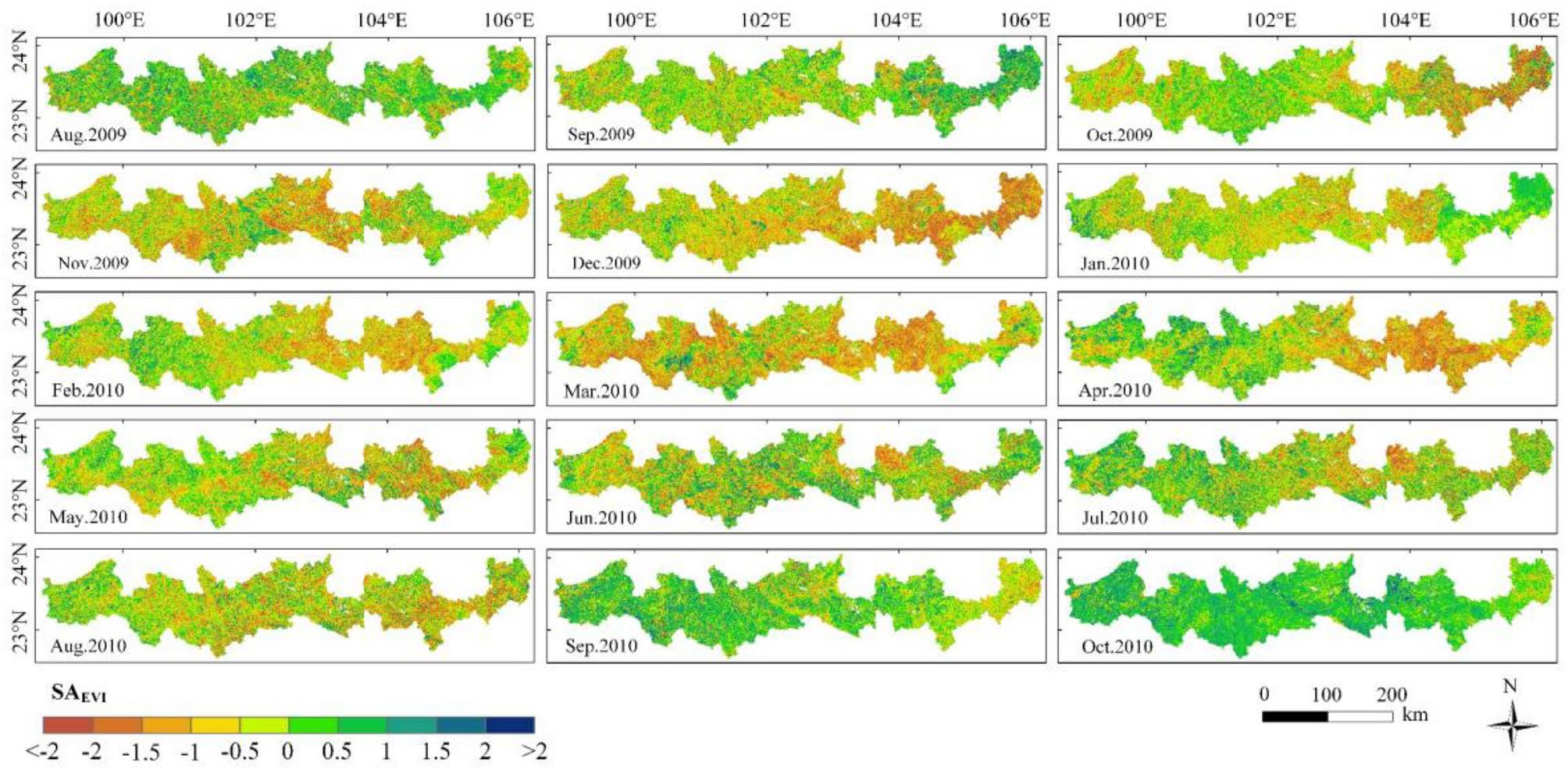
Spatial distribution of SA_EVI_ from August 2009 to October 2010—created using ArcGIS v10.2^22^.

Figure 6 shows the spatial distribution of SA_EVI_ in the study area from August 2009 to October 2010. This drought event obviously had an inhibitory effect on the EVI. Prior to October 2009, SA_EVI_ was negative only in some hot-dry river valley areas, covering less than 40% of the area. In October, SA_EVI_ was negative in the eastern region and the western valley region. In November, SA_EVI_ in the eastern region and western valley region showed a large area of negative values, when the area reached 54.92%. The months with the largest negative area of SA_EVI_ were December 2009 and March 2010, which were 65.94% and 65.68%, respectively, and the corresponding SA_EVI_ averages for these two months were − 0.77 and − 0.76, respectively. However, in January and February 2010, only 43.28% and 49.68% of SA_EVI_ areas were negative, respectively, which may be influenced by vegetation phenology. From January to February, vegetation was in a dormant period. Compared with the growth of vegetation in normal years, there was little difference in the effect of drought on vegetation in these two months. From March onwards, vegetation began to grow, and the vegetation suffering from drought showed a large difference from the vegetation in normal years. After July 2010, with the decrease in drought degree, the vegetation in the study area gradually recovered, and the area with negative SA_EVI_ was less than 40%. By October 2010, the area with a negative SA_EVI_ had decreased to 6.52%, indicating that most vegetation had recovered from the drought.

#### Analysis of the LAI standardized anomaly index

**Figure 7:**
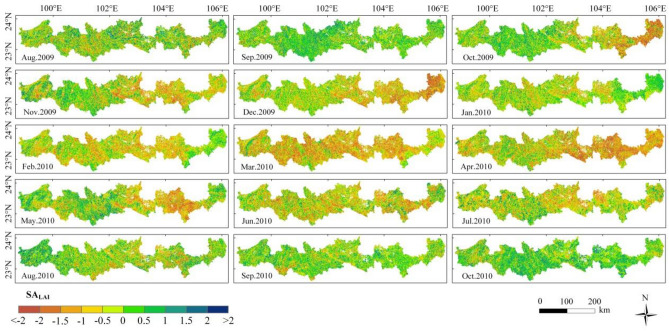
Spatial distribution of SA_LAI_ from August 2009 to October 2010—created using ArcGIS v10.2^22^.

The spatial distribution and temporal variation of SA_LAI_ are similar to those of SA_EVI_ (Fig. 7), but there were also a few differences with SA_EVI_, the area of SA_LAI_ with a negative value dropped to 18.58% in October, which was higher than the area of SA_EVI_ with a negative value, indicating that compared with EVI, some LAI had not recovered at that time.

#### Analysis of the GPP standardized anomaly index

**Figure 8:**
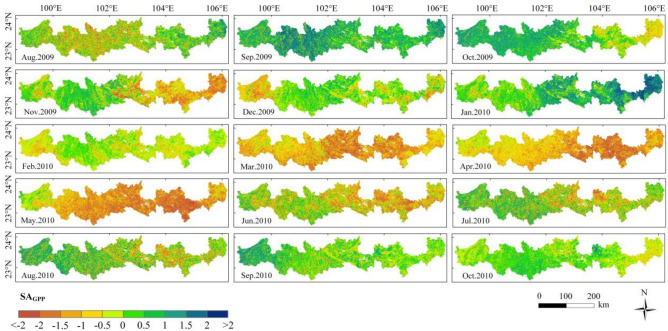
Spatial distribution of SA_GPP_ from August 2009 to October 2010—created using ArcGIS v10.2^22^.

Figure 8 shows the spatial distribution of SA_GPP_, which was different from that of SA_EVI_ and SA_LAI_. From March 2010 to May 2010, SA_GPP_ showed a wide range of obvious negative anomalies, with the area of negative anomalies reaching more than 85%, and the area of extreme negative anomalies (SA_GPP_ < − 2) was larger. The areas of extreme negative anomalies in EVI and LAI were 4.74% and 5.13%, respectively, while the area of GPP reached 10.95%.

In summary, EVI, LAI and GPP all showed a positive impact on drought events, but GPP showed an obvious response to drought events. From the perspective of spatial distribution characteristics, GPP showed the strongest aggregation of vegetation negative anomaly areas, while EVI and LAI showed weak aggregation of vegetation negative anomaly areas.

From the perspective of spatial distribution, vegetation in the eastern region is more significantly affected by drought compared to the western region. We know that drought is typically triggered by dynamic interactions between the atmosphere and the Earth's surface, which can alter water fluxes over extended periods, such as precipitation, evaporation, and transpiration. The main reasons for the more pronounced drought in the eastern region compared to the western region are less precipitation and less surface water. The eastern region is located on the leeward slope of the Ailao Mountains, where natural barriers reduce precipitation. At the same time, the eastern region is characterized by a distribution of karst landforms, with poor and thin soil layers. Precipitation quickly infiltrates underground, reducing surface water supply, exacerbating surface drought, and leading to a more fragile ecological environment. Therefore, the drought resistance of vegetation in these areas is weaker. This has been confirmed by existing research^29–31^.

### Analysis of vegetation recovery time

**Figure 9:**
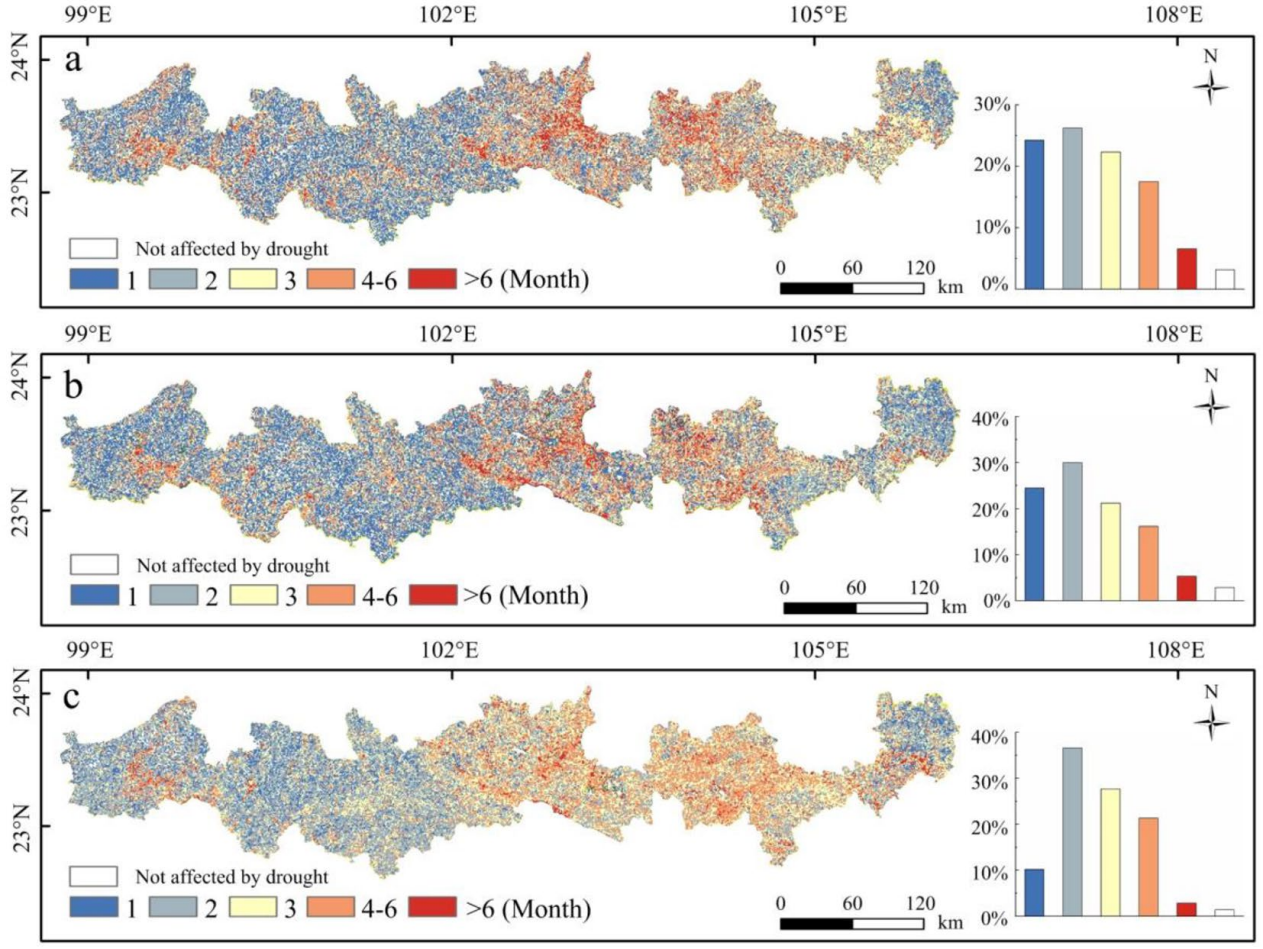
Spatial distribution of time needed for vegetation restoration and the proportion of each time (**a**. EVI; **b**. LAI; **c**. GPP—created using ArcGIS v10.2^22^ and Microsoft Office Excel v2016^32^.

Figure 9 shows the time needed for EVI, LAI and GPP to recover to normal levels after drought, as well as their spatial distributions. These three indicators show that more than 95% of the area's vegetation has been affected by drought. The areas where vegetation recovery took 1 to 2 months had recovery rates of 50% for EVI, 54% for LAI, and 47% for GPP. These regions are primarily located in the eastern part of the study area in Funing County and the western part of the longitudinal valley region, which experience relatively mild drought conditions, and vegetation growth is lush. The areas where vegetation recovery took 3 months had recovery proportions of 22.76% for EVI, 20.41% for LAI, and 28.64% for GPP. These regions are scattered throughout the study area, with no distinct distribution patterns. The proportions of EVI, LAI and GPP recovered after 4 to 6 months were 17.51%, 16.17% and 21.34%, respectively. The proportions of EVI, LAI and GPP recovered after more than 6 months were 6.57%, 5.34% and 2.87%, respectively. The above areas are located in the central and eastern parts of the study area where drought is most severe.

## Discussion

### Influence of temperature and precipitation on this drought event

**Figure 10:**
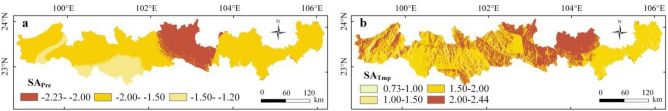
Spatial distribution of the maximum SA_Tmp_ and minimum SA_Pre_ from May 2009 to December 2010—created using ArcGIS v10.2^22^.

Figure 10 shows the maximum SA_Tmp_ and minimum SA_Pre_ values in the study area from May 2009 to December 2010. As shown in Fig. 10, there were obvious negative precipitation anomalies and positive temperature anomalies, indicating that reheat stress exists in the study area in addition to water shortages. Among them, the most obvious negative precipitation anomalies were concentrated in Shiping County, Jianshui County and Gejiu city, while the positive temperature anomalies were concentrated in Yanshan County, Wenshan County, Mengzi County and Jianshui County; these areas basically correspond to the areas with extreme drought. The spatial heterogeneity of this drought event is closely related to temperature and precipitation, which is consistent with the results of Dong et al^33^.

### Difference of response of vegetation indexes to drought

**Figure 11:**
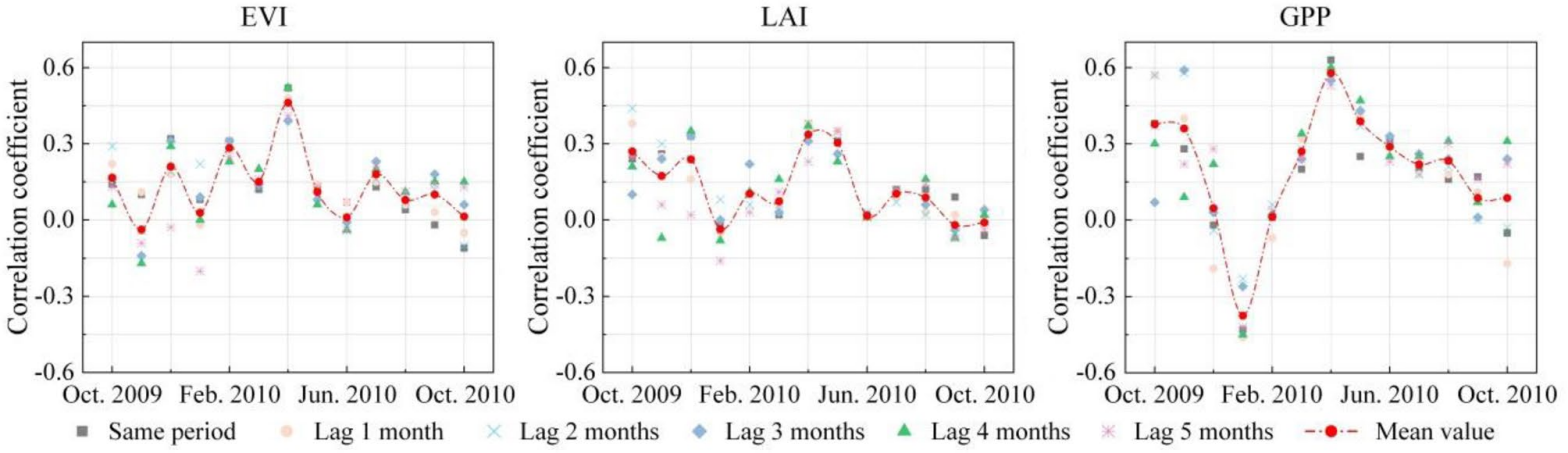
Correlation analysis between vegetation index and drought.

Figure 11 shows the correlation coefficients of SA_SPEI-3_ with SA_EVI_, SA_LAI_ and SA_GPP_. From the correlation coefficient between SA_EVI_, SA_LAI_, and SA_GPP_ with SA_SPEI-3_, except for December 2009 to February 2010, SA_GPP_ and SA_SPEI-3_ had the highest correlation coefficient in other months, followed by EVI, and the maximum correlation coefficient of LAI was lower than the other two. The reason is that the LAI index quickly becomes saturated in forest areas with high vegetation coverage, which makes the index insensitive to small changes in leaf biomass. This result is consistent with the research results of Vicca S^34^.

From the month corresponding to the maximum correlation coefficient, the strongest correlation between SA_EVI_, SA_LAI_ and SA_GPP_ with SA_SPEI-3_ was in April 2010, followed by February, May and May 2010. In other words, the growth of vegetation in spring was more susceptible to drought. As seen from Sect. 4.1, the drought from October 2009 to March 2010 was relatively strong, and after nearly six months of drought accumulation, the surface water shortage was very serious. At the same time, spring is the greening season for vegetation. Affected by drought, vegetation does not have enough water to develop new leaves, which makes the impact of drought on vegetation in spring more obvious.

It can be observed from Fig. 11 that EVI and LAI show higher proportions of recovery within 1–2 months, while GPP exhibits a higher proportion of recovery within 2–3 months. Furthermore, the proportion of GPP recovery exceeding 6 months was lower than that of EVI and LAI. This is mainly attributed to the lagged response of the EVI and LAI to drought. The greenness and canopy biomass of vegetation are not immediate results of current photosynthesis but rather represent the cumulative photosynthetic yield over an extended period. They do not immediately respond to changes in thermal factors^35^. The short-term recovery of vegetation is because the current drought did not cause significant damage to EVI and LAI within the short term, but it had a noticeable impact on vegetation GPP. Additionally, GPP is sensitive to changes in water availability. When the water supply improves, vegetation can recover its photosynthetic activity relatively quickly, transitioning from a drought state.

### Differences in the degree and the lag of response to drought among different vegetation types

**Figure 12:**
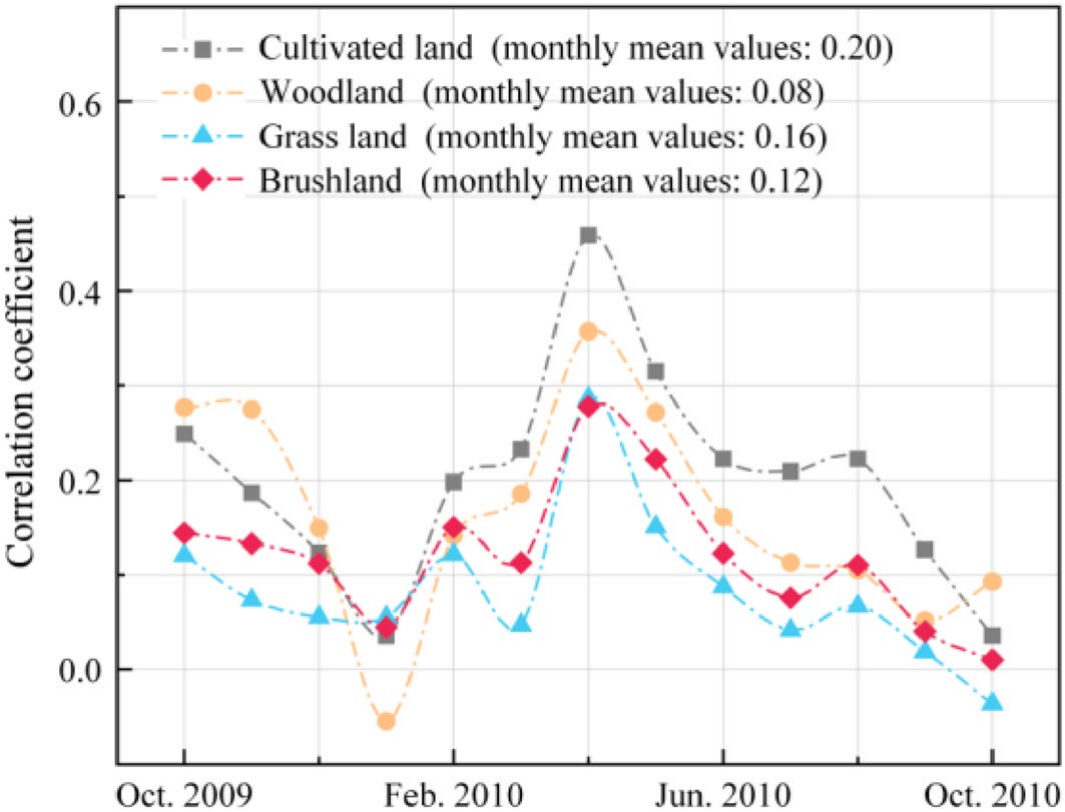
Correlation analysis between vegetation index and drought of different vegetation types.

According to the correlation coefficients of SA_EVI_, SA_LAI_, SA_GPP_ and SA_SPEI-3_ (Fig. 12), drought has different impacts on different vegetation types. The correlation coefficient of cultivated land was the highest, indicating that cultivated land was more susceptible to drought, which was consistent with previous research results^36–38^. The correlation of forestland was second, and that of shrub land was third. The correlation between grassland and drought index was the lowest, which may be related to the particularity of the study area. Grassland in the study area mostly grows in the karst landform area, which has low vegetation coverage and a fragile ecosystem^39,40^. There was little difference in the growth status of grassland in normal years and drought years.

**Figure 13:**
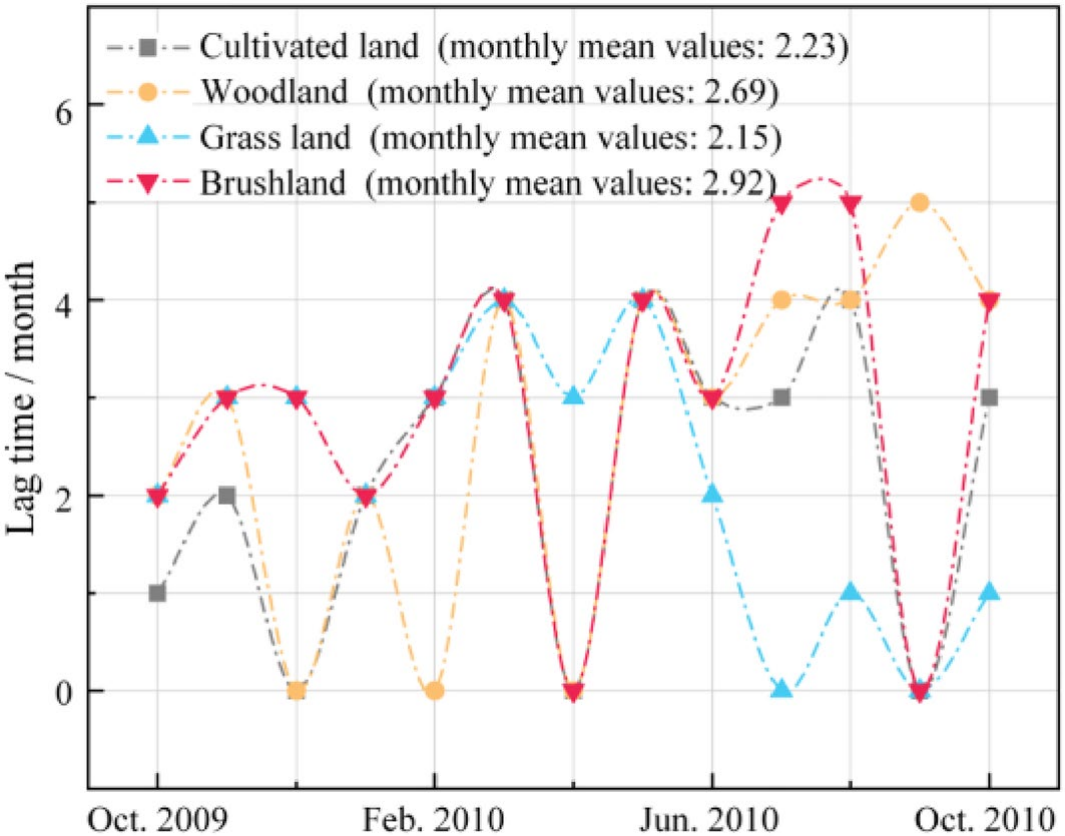
Lag months of response of different vegetation types to drought.

For the lag time (Fig. 13), the lag time of shrubland was the longest (2.92 months), followed by forestland (2.69 months), cultivated land (2.23 months), and grassland (2.15 months). This is consistent with previous research results^41,42^. Compared with grassland and cultivated land, forestland and shrubland have deeper roots, and vegetation can obtain water from deep soil after drought occurs and have stronger drought tolerance^15,43,44^. In contrast, grassland has a simple structure and shallow root system, which will cause the upper part to wither faster after drought. Although cultivated land is similar to grassland, it is subject to artificial control measures, such as irrigation, and will respond to drought slightly later than grassland.

### Analysis of the time needed for the restoration of different vegetation types

**Figure 14:**
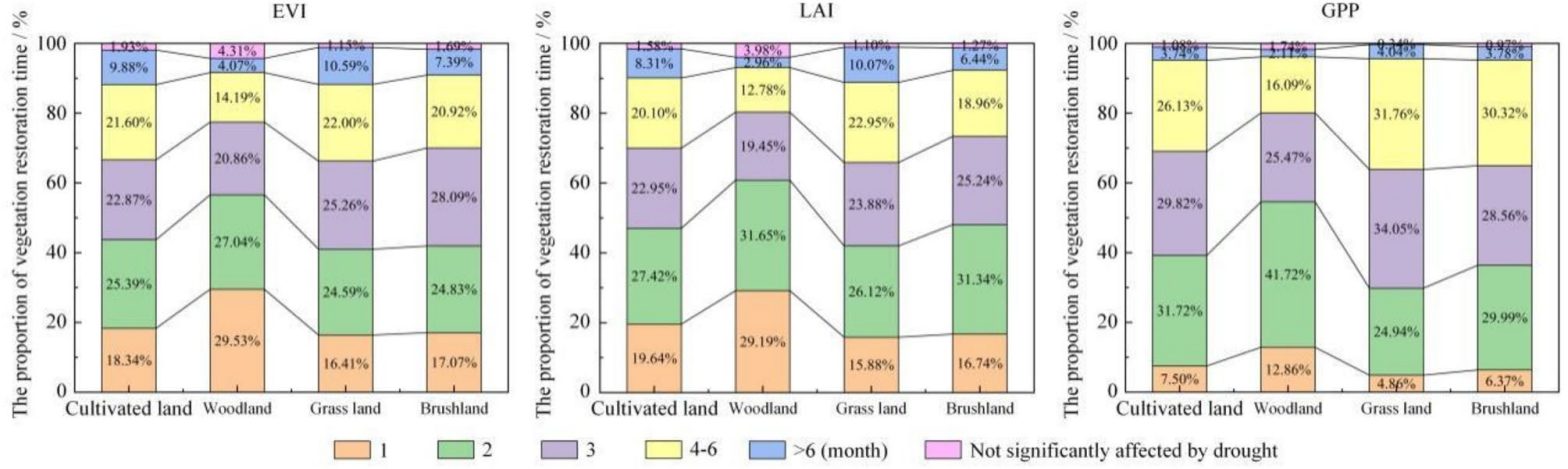
The proportion of time needed for the restoration of different vegetation types.

The recovery time of different vegetation types was different (Fig. 14). The EVI, LAI and GPP indices showed that the recovery of forestland was the fastest, followed by shrub land, cultivated land, and grassland. The recovery ratios of EVI, LAI and GPP reached 91.62%, 93.06% and 96.15%, respectively, in forestland within 6 months and 90.91%, 92.29% and 95.24% in shrubland within 6 months. In contrast, the recovery time of cultivated land and grassland was longer, with more than 8% of cultivated land and 10% of grassland not recovering EVI and LAI index within 6 months. The recovery time of cultivated land is lower than that of grassland, which is obviously due to artificial agricultural management measures, which promote the recovery of cultivated land^45,46^.

Generally, the structure of grassland is simple, and it will recover quickly after the end of drought, but the results of this paper show that the recovery time of grassland is the longest because the grassland is mainly distributed in the central and eastern regions, where the drought degree is severe and the drought duration is long, resulting in the delayed recovery of grassland in the drought. The recovery of forestland is the fastest for the following reasons. First, the forestland is mainly distributed in the western longitudinal mountain area with a low drought degree and short drought duration, and the vegetation is less affected by drought. Second, when drought occurs, the forest will maintain basic metabolic capacity by adjusting stomatal openness based on the water deficit to avoid excessive water loss caused by drought, and the forest itself has strong water-locking and drought resistance^18,47^. When drought ends, forests that are not obviously affected by drought will recover quickly.

## Conclusions

The study takes the extreme drought event in the Tropic of Cancer (Yunnan section) during 2009–2010 as an example to analyse the response of the vegetation ecosystem to this drought event. We selected SPEI-3 as the drought index and EVI, LAI and GPP as the vegetation indices. Various methods, including standardized anomaly calculation, correlation index analysis, and specific threshold extraction, were employed to comprehensively analyse the characteristics of this drought event, the responses of different vegetation indices to drought, and the duration needed for vegetation recovery.

The conclusions are as follows: (1) Due to the cumulative effects of drought and vegetation phenology, the most severely affected months for vegetation were March to May 2010. (2) EVI, LAI, and GPP exhibit differential responses to drought. GPP can more accurately identify the regions where drought impacts vegetation. (3) Following the extreme drought event, 70% of the vegetation recovered within 3 months, while a small amount of vegetation still had not recovered after 6 months. (4) Different vegetation types exhibit distinct responses to drought. Croplands and grasslands show the strongest response to drought with shorter lag times but longer recovery periods. Forests and shrublands, on the other hand, exhibit weaker responses to drought, longer lag times, and shorter recovery periods.

The main focus of this study is to analyse the impact of this drought event on vegetation using the SPEI calculated from temperature and precipitation. These two factors are the primary meteorological elements affecting vegetation growth. However, other meteorological factors, such as sunshine duration, relative humidity, and evaporation, can also influence vegetation growth. Subsequent research will attempt to incorporate additional climate factors to further refine the mechanistic analysis of how climate factors affect vegetation.

## Data Availability

The datasets generated and/or analysed during the current study are not publicly available due the county-level meteorological data is not public data in China, but are available from the corresponding author on reasonable request.

## References

[1] Summary for Policymaker. IPCC Climate Change 2021: The Physical Science Basin (2021).

[2] Reichstein M (2013). Climate extremes and the carbon cycle. Nature.

[3] Allen CD (2010). A global overview of drought and heat-induced tree mortality reveals emerging climate change risks for forests. For. Ecol. Manag..

[4] Barbeta A (2015). The combined effects of a long-term experimental drought and an extreme drought on the use of plant-water sources in a Mediterranean forest. Glob. Chang. Biol..

[5] Piao S (2019). The impacts of climate extremes on the terrestrial carbon cycle: A review. Sci. China Earth Sci..

[6] Environmental Protection Department of Yunnan Province, Kunming Institute of Botany, Yunnan University, et al. Ecosystem List of Yunnan Province (2018), China (2018).

[7] Myers N, Mittermeier RA, Fonseca GAB, Kent J (2000). Biodiversity hotspots for conservation priorities. Nature.

[8] Olson DM, Dinerstein E (1998). The global 200: A representation approach to conserving the earth’s most biologically valuable ecoregions. Conserv. Biol..

[9] Han Y, Jiang XJ (2018). Analysis on drought characteristics in Yunnan Province and determination of typical drought year. Water Resources Dev. Manag..

[10] Liu J, Tan X, Wan J, Ma J, Zhang N (2011). Comparative analysis between the 2010 severe drought in southwest China and typical drought disasters. Chin. Water Resour..

[11] Tan, Y. Y. Study on drought management mode of crops in Yunnan Province based on risk management. M.S. thesis, Fudan University (2015).

[12] Wang, C. J. Geographical distribution pattern and spatial conservation prioritization for wild plants in China under climate chang. M.S. thesis, Beijing forestry University (2020).

[13] Han, Q. H. Recent patterns of algal and carbon cycling changes in response to climate change and catchment development in two medium-sized reservoirs in Southeast Yunnan. M.S. thesis, Yunnan Normal University (2021).

[14] Chen S, Huang Y, Wang G (2020). Detecting drought-induced GPP spatiotemporal variabilities with sun-induced chlorophyll fluorescence during the 2009/2010 droughts in China. Ecol. Indic..

[15] Li X (2019). The impact of the 2009/2010 drought on vegetation growth and terrestrial carbon balance in Southwest China. Agric. For. Meteorol..

[16] Song L (2019). Divergent vegetation responses to extreme spring and summer droughts in Southwestern China. Agric. For. Meteorol..

[17] Zhao Z, Wu X, Li G, Li J (2015). Drought in southwestern China and its impact on the net primary productivity of vegetation from 2009–2011. Acta Ecol. Sin..

[18] Zhang X, Yamaguchi Y, Li F, He B, Chen Y (2017). Assessing the Impacts of the 2009/2010 drought on vegetation indices, normalized difference water index, and land surface temperature in Southwestern China. Adv. Meteorol..

[19] Zhou L, Wang SQ, Chi YG, Wang JB (2018). Drought impacts on vegetation indices and productivity of terrestrial ecosystems in southwestern china during 2001–2012. Chin. Geogr. Sci..

[20] Zhang Y, Wang J, Su H, Cheng F (2021). Study on landform classification of dual-scale watershed units based on CART: A Case of study of the tropic of cancer (Yunnan Section). Geogr. Geo-inf. Sci..

[21] Zhang Y, Wang J, Nong L, Cheng F, Zhang Y (2021). Spatio-temporal variation of vegetation phenology and its response to climate in the tropic of cancer (Yunnan section) based on MODIS time-series data. Ecol. Environ. Sci..

[22] ESRI ArcGIS Desktop v10.2, URL: https://www.esri.com/en-us/home, [Software] (2013).

[23] Vicente-Serrano SM, Beguería S, López-Moreno JI (2010). A multiscalar drought index sensitive to global warming: The standardized precipitation evapotranspiration index. J. Clim..

[24] Fang L, Wang D, Fang G (2019). Analysis of drought trend in arid zone of central Ningxia based on standardized precipitation evapotranspiration index. Sci. Technol. Eng..

[25] Saft M, Western AW, Zhang L, Peel MC, Potter NJ (2015). The influence of multiyear drought on the annual rainfall-runoff relationship: An A ustralian perspective. Water Resour. Res..

[26] Pei F, Li X, Liu X, Lao C (2013). Assessing the impacts of droughts on net primary productivity in China. J. Environ. Manag..

[27] Zhao L (2014). Impacts of meteorological drought on net primary productivity of forest in Hubei Province. Resources Environ. Yangtze Basin.

[28] Gu X (2021). Assessment of the cumulative and lagging effects of drought on vegetation growth in Inner Mongolia. Acta Agrestia Sinica.

[29] Pekel JF, Cottam A, Gorelick N, Belward AS (2016). High-resolution mapping of global surface water and its long-term changes. Nature.

[30] Zhao M, Geruo A, Velicogna I, Kimball JS (2017). Satellite observations of regional drought severity in the continental united states using GRACE-based terrestrial water storage changes. J. Clim..

[31] Li, Z. Spatial-temporal variation characteristics and influencing factors of drought in karst region based on Multi-source Data. M.S. thesis, Guilin University of Technology (2023).

[32] Microsoft Office Excel v2016, URL: https://www.microsoft.com/zh-cn/download/office, [Software] (2015).

[33] Dong B, Yu Y, Wu X (2022). The drought legacy effects of 2009–2010 based on NDVI in Yunnan province. Acta Ecologica Sinica.

[34] Vicca S (2016). Remotely-sensed detection of effects of extreme droughts on gross primary production. Sci. Rep..

[35] Wagle P (2014). Sensitivity of vegetation indices and gross primary production of tallgrass prairie to severe drought. Remote Sens. Environ..

[36] Ciais P (2005). Europe-wide reduction in primary productivity caused by the heat and drought in 2003. Nature.

[37] Lesk C, Rowhani P, Ramankutty N (2016). Influence of extreme weather disasters on global crop production. Nature.

[38] Schwalm CR (2010). Assimilation exceeds respiration sensitivity to drought: A fluxnet synthesis. Glob. Chang. Biol..

[39] Heilman JL (2014). Water-storage capacity controls energy partitioning and water use in karst ecosystems on the Edwards Plateau, Texas. Ecohydrology.

[40] Liu M, Xu X, Wang D, Sun AY, Wang K (2016). Karst catchments exhibited higher degradation stress from climate change than the non-karst catchments in southwest China: An ecohydrological perspective. J. Hydrol..

[41] Teuling AJ (2010). Contrasting response of European forest and grassland energy exchange to heatwaves. Nat. Geosci..

[42] Wolf S (2016). Warm spring reduced carbon cycle impact of the 2012 US summer drought. Proc. Natl. Acad. Sci. USA.

[43] Deng Y (2021). Responses of vegetation greenness and carbon cycle to extreme droughts in China. Agric. For. Meteorol..

[44] Xu C (2019). Increasing impacts of extreme droughts on vegetation productivity under climate change. Nat. Clim. Chang..

[45] Fan M (2007). Nitrogen input, 15N balance and mineral N dynamics in a rice–wheat rotation in southwest China. Nutr. Cycl. Agroecosyst..

[46] Jiang Z, Lian Y, Qin X (2014). Rocky desertification in southwest china: Impacts, causes, and restoration. J. Earth-Sci. Rev..

[47] Giardina F (2018). Tall Amazonian forests are less sensitive to precipitation variability. Nat. Geosci..

